# Identifying key bird species and geographical hotspots of avian influenza A (H7N9) virus in China

**DOI:** 10.1186/s40249-018-0480-x

**Published:** 2018-10-11

**Authors:** Benyun Shi, Xiao-Ming Zhan, Jin-Xin Zheng, Hongjun Qiu, Dan Liang, Yan-Ming Ye, Guo-Jing Yang, Yang Liu, Jiming Liu

**Affiliations:** 1 School of Cyberspace, Hangzhou Dianzi University, Hangzhou, 310018 People’s Republic of China; 2 School of Computer Science and Technology, Hangzhou Dianzi University, Hangzhou, 310018 People’s Republic of China; 3 Jiangsu Institute of Parasitic Diseases, Wuxi, 214064 People’s Republic of China; 4 Department of Epidemiology and Public Healthy, Swiss Tropical and Public Health Institute, Basel, Switzerland; 5 University of Basel, Basel, Switzerland; 6 State Key Laboratory of Biocontrol, Department of Ecology and School of Life Sciences, Sun Yat-Sen University, Guangzhou, 510275 People’s Republic of China; 7 Department of Computer Science, Hong Kong Baptist University, Kowloon Tong, Hong Kong, People’s Republic of China

**Keywords:** Avian influenza virus, Bird migration, Geographical hotspots, Phylogenetic analysis, Cross correlation function

## Abstract

**Background:**

In China since the first human infection of avian influenza A (H7N9) virus was identified in 2013, it has caused serious public health concerns due to its wide spread and high mortality rate. Evidence shows that bird migration plays an essential role in global spread of avian influenza viruses. Accordingly, in this paper, we aim to identify key bird species and geographical hotspots that are relevant to the transmission of avian influenza A (H7N9) virus in China.

**Methods:**

We first conducted phylogenetic analysis on 626 viral sequences of avian influenza A (H7N9) virus isolated in chicken, which were collected from the Global Initiative on Sharing All Influenza Data (GISAID), to reveal geographical spread and molecular evolution of the virus in China. Then, we adopted the cross correlation function (CCF) to explore the relationship between the identified influenza A (H7N9) cases and the spatiotemporal distribution of migratory birds. Here, the spatiotemporal distribution of bird species was generated based on bird observation data collected from China Bird Reports, which consists of 157 272 observation records about 1145 bird species. Finally, we employed a kernel density estimator to identify geographical hotspots of bird habitat/stopover that are relevant to the influenza A (H7N9) infections.

**Results:**

Phylogenetic analysis reveals the evolutionary and geographical patterns of influenza A (H7N9) infections, where cases in the same or nearby municipality/provinces are clustered together with small evolutionary differences. Moreover, three epidemic waves in chicken along the East Asian–Australasian flyway in China are distinguished from the phylogenetic tree. The CCF analysis identifies possible migratory bird species that are relevant to the influenza A(H7N9) infections in Shanghai, Jiangsu, Zhejiang, Fujian, Jiangxi, and Guangdong in China, where the six municipality/provinces account for 91.2% of the total number of isolated H7N9 cases in chicken in GISAID. Based on the spatial distribution of identified bird species, geographical hotspots are further estimated and illustrated within these typical municipality/provinces.

**Conclusions:**

In this paper, we have identified key bird species and geographical hotspots that are relevant to the spread of influenza A (H7N9) virus. The results and findings could provide sentinel signal and evidence for active surveillance, as well as strategic control of influenza A (H7N9) transmission in China.

**Electronic supplementary material:**

The online version of this article (10.1186/s40249-018-0480-x) contains supplementary material, which is available to authorized users.

## Multilingual abstracts

Please see Additional file 1 for translations of the abstract into the five official working languages of the United Nations.

## Background

The geographical spread of avian influenza viruses (AIVs) has been and will continue to be a serious public health concern in China. Since February 2013, the influenza A (H7N9) with a high mortality rate in humans has been spreading in the Yangtze River Delta and is still prevalent in Eastern China till now [1–4]. Evidence shows that bird migration plays an essential role in the global spread of AIVs [5–7]. As one of the three major flyways for bird migration that pass by China (i.e., the Central Asian, East Asian-Australasian and West Pacific migratory bird flyways), the risk of avian influenza spreading in Eastern China, including the Yangtze River Delta, the Pearl River Delta, is especially high. Although great efforts have been made to investigate the global spread of AIVs based on intercontinental flyways of migratory birds [8–13], one of the fundamental challenges is to investigate the roles played by migratory birds in the regional/provincial short-distance movement.

As a natural reservoir of AIVs, birds in wetlands and aquatic environments, such as Anseriformes and Charadriiformes, harbour the major avian influenza viruses [14, 15]. Many bird species may share the same habitats or stopovers during their migration, while effective transmissions are more likely to happen through the faecal-oral route via surface waters [16]. Active surveillance of bird infections could provide “early-warning” for the introduction of AIVs into new regions. To combat the growing threat of bird flu, many studies have been conducted to estimate the prevalence of various AIVs in different bird species and localities. For example, Olsen et al. have reviewed the global prevalence of influenza A viruses in wild birds, such as ducks, gulls, terns, and waders [17]. Pawar et al. have estimated the prevalence of H5N1 in wild birds in India [18]. Bi et al. have collected and isolated influenza H5N1 viruses from sick or dead birds in the Sanmenxia Reservoir Area of China in 2015 [19]. However, there is still a lack of systematic research on the influenza A (H7N9) virus with respect to all bird species across the country.

In 2005, a global network for AIVs among wild birds nationally and internationally was appealed to the United States Congress to promote the worldwide surveillance. Since then, many genetic sequence databases, such as the Global Initiative on Sharing All Influenza Data (GISAID, https://www.gisaid.org/), have been designed to encourage the sharing of all influenza type viral sequences. In doing so, phylogenetic analysis can be implemented to uncover genomic characterization and molecular evolution of the circulating AIVs [20–23]. Based on the coalescent theory [24, 25], the demographic history of a host population, such as the effective population size, can further be reconstructed from a phylogenetic tree by assuming different parametric models on the population dynamics [26–30]. More importantly, with the high throughput sequencing technology, it would also be possible to integrate genetic variability and evolution of AIVs with virus-host ecology (e.g., migratory birds). Along this line, many phylogeographic studies have been conducted to analyse the relationship between global spread of AIVs and migration flyways of migratory birds [10, 31–33]. However, because most of the isolated viral sequences in GISAID are annotated without precise geographical location and specific virus-host information, such studies can only be implemented at a coarse-grained scale.

In this paper, we aim to identify the possible bird species and geographical hotspots that are relevant to the spread of influenza A (H7N9) at a finer-grained scale in China. First, we collect all gene sequences of H7N9 from GISAID, which are isolated in chicken in China from January 1, 2013 to December 31, 2017. Accordingly, phylogenetic trees are constructed using the MEGA software to explore their evolutionary relationship in terms of geographical locations. Then, based on 157 272 observation data of 1145 bird species in China, we adopt the cross correlation function (CCF) to investigate the relationship between the identified influenza A (H7N9) cases in chicken and the spatiotemporal distribution of migratory bird species. In doing so, we identify a list of possible bird species that are relevant to the isolated influenza A (H7N9) cases in six municipality/provinces with high incidences. Finally, we explore and visualize geographical hotspots of those identified bird species. Both the identified bird species and their geographical distribution would provide sentinel signal and evidence for the implementation of active surveillance in bird flu intervention and control.

## Methods

### Data collection and pre-processing

Full- or partial- length hemagglutinin (HA) and neuraminidase (NA) sequences of influenza A (H7N9) virus isolated in chicken in China were collected from the Global Initiative on Sharing All Influenza Data (GISAID) from January 1, 2013 to December 31, 2017. Each sequence was associated with an isolated ID, and annotated with a location (i.e., municipality or province) and the date of isolation. In this paper, we adopted HA and NA subtypes with lengths greater than or equal to 1683 and 1398, respectively, to construct the phylogenetic trees. After removing duplicated sequences, 495 sequences remained for further analysis. All the 495 aligned sequences were included in Additional file 2.

The bird observation data was collected from a citizen science project, where thousands of bird-watching enthusiasts and experts share their observations through an online forum. Totally, there are 1145 bird species observed in China. Each record is about one bird species, which includes the scientific name, the locality (i.e., the longitude and latitude), the date of observation, the number of observed birds and the observer’s name. All observation records were checked by bird experts based on the biological nature of each bird species, and then published in China Bird Report annually. Since many observers may watch birds at the same location and date, in this case, only one record with the largest number of observed birds was retained, and all other reduplicated records are filtered out from the dataset. After removing duplications, there remained 157 272 observation records during the year 2008 and 2009. Further, with the help of bird experts, we selected 150 common migratory birds from the totally 1145 bird species for CCF analysis (see Additional file 3). All observation data were spatially aggregated by municipality/provinces, and temporally aggregated by weeks in a year.

### Phylogenetic analysis

The HA and NA segments of selected influenza A (H7N9) virus were first aligned by CLUSTAL W algorithm implemented in Clustal v.2.1 (http://www.clustal.org/download/current/) [34]. Then, the HA and NA fragments were intercepted to be the same length in BioEdit v.7.0.5 (http://www.mbio.ncsu.edu/bioedit/bioedit.html) [35], and spliced in MEGA v.6.0 (https://www.megasoftware.net/mega.php) [36]. Phylogenetic trees of the spliced HA and NA sequences were constructed using the neighbour-joining (NJ), maximum parsimony (MP), and maximum likelihood (ML) approaches. The nucleotide substitution model was determined using Akaike Information Criterion (AIC) in jModelTest v.2.1.10 (https://github.com/ddarriba/jmodeltest2) [37]. With respect to our dataset, the General Time Reversible model assuming a rate variation across sites according to a gamma-shaped distribution with invariant sites was selected. For the NJ approach, the composite maximum likelihood algorithms were used to estimate the transversion/transition bias and the nucleotide substitution patterns. For the ML approach, the heuristic searching strategy for the best topology was started via five random BioNJ trees, and those trees were moved by nearest-neighbour interchange. Tree reliabilities were tested with 1000 bootstrap replicates to yield a majority consensus tree. To clearly demonstrate the reconstructed phylogenetic tree in this paper, branches with bootstrap values less than 0.6 were filtered out. Then, the remaining 184 gene sequences (see Additional file 4 for detail) were reconstructed using the NJ, MP, and ML approaches, respectively. Moreover, a date-calibrated tree is also generated to reveal the epidemic waves of influenza A (H7N9). Finally, the NJ tree was visualized, edited and coloured in FigTree v.1.4.3 (https://www.megasoftware.net/mega.php) and iTOL (iTOL: http://itol.embl.de/).

### Cross correlation analysis

In statistics, cross correlation was used for measuring of the similarity between two series as a function of the displacement of one relative to the other. In this paper, the sample cross correlation function (CCF) was adopted to identify lags of observed migratory birds that might be useful predictors of influenza A (H7N9) incidences. A positive *Lag* value represented the correlation between the amount of observed bird species at time *t* and the number of influenza A (H7N9) cases at time *t* + *Lag*. The CCF command in R software (https://www.r-project.org/) was *ccf*(*x*, *y*, *Lag*), where *x* and *y* represent time series of H7N9 cases and migratory birds, respectively. Specifically, the CCF analysis was implemented to analyse bird observation data collected from Shanghai and five provinces (Jiangsu, Zhejiang, Fujian, Jiangxi, and Guangdong) with high H7N9 infections. One reason to select these six municipality/provinces was that they are geographically close to each other in Eastern China, and the number of identified H7N9 cases in these areas accounted for 91.2% of the total number of isolated cases in chicken in China based on the collected data from GISAID. Therefore, it would be helpful to investigate the possible bird species and hotspots in a finer-grained scale in these areas for the implementation of active surveillance on the potential epidemics of influenza A (H7N9). The identified hotspots in specific municipality/province were illustrated using the kernel method in ArcGIS v10.5 (Environmental Systems Research Institute, Inc., RedLands, California, USA).

## Results

**Fig. 1:**
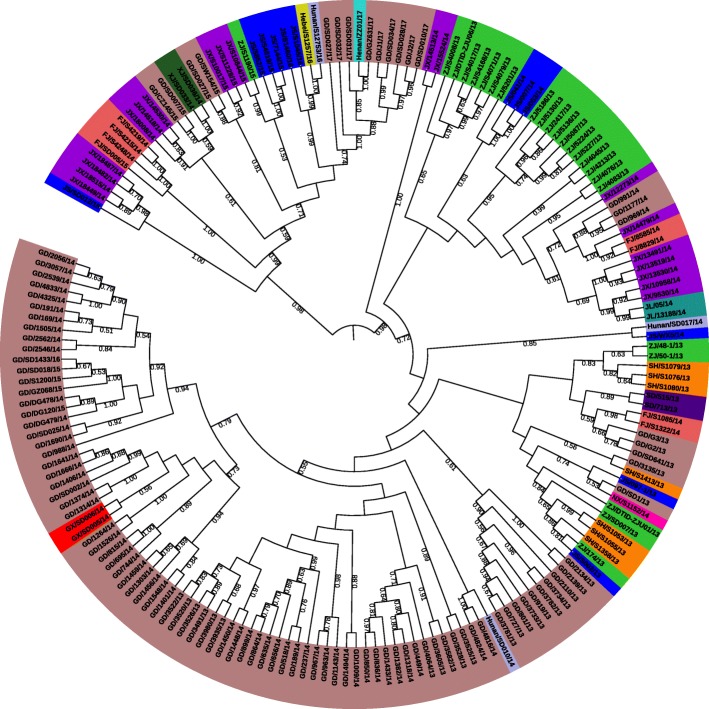
Phylogenetic tree reconstructed by neighbour joining (NJ) approach based on 184 HA and NA segments of avian influenza A (H7N9) virus. Each leaf is labelled with the name abbreviation of a sequence, where the first two letters stand for the isolated location, and the last two numbers stand for the isolated year. Sequences isolated from the same municipality/provinces are marked with the same colour. Bootstrap values greater than 0.5 are marked at the branches

Figure 1 demonstrates the phylogenetic tree constructed using the NJ approach based on 184 intercepted HA and NA segments of influenza A (H7N9) virus in China during January 1, 2013 to December 31, 2017. Each leaf is labelled with the name abbreviation of a sequence, where the first two letters stand for the isolated municipality/province, and the last two numbers stand for the year of isolation. The sequences isolated from the same municipality/provinces are marked with the same colour. Bootstrap values greater than 0.5 are shown at the branches. It can be observed that most sequences isolated at the same municipality/province and in the same year are clustered together (e.g., sequences isolated in Guangdong with brown colour in Fig. 1). Note that in this paper, we only use the NJ tree for illustration, similar results can be obtained using MP and ML approaches (see Additional files 5 and 6).

With respect to geographical spread of influenza A (H7N9) virus, the phylogenetic tree in Fig. 1 (and the date-calibrated tree in Additional file 7) reveals that there exist three major epidemic waves in chicken in Southeast China. The first wave happened mainly in Zhejiang and Jiangsu (marked in green and blue at the top-right corner in Fig. 1) in the spring of 2013. After that, the virus gradually spread to Southern China (i.e., Jiangxi, Fujian, and Guangdong), and broke out in Guangdong in 2014. While the third wave happened in almost all provinces in Eastern China from 2014 to 2017 (see subtree at the top-left corner in Fig. 1), and potentially new strain of H7N9 virus emerged. These observations are consistent with Liu et al.‘s findings about human infections of influenza A (H7N9) virus [38].

**Fig. 2:**
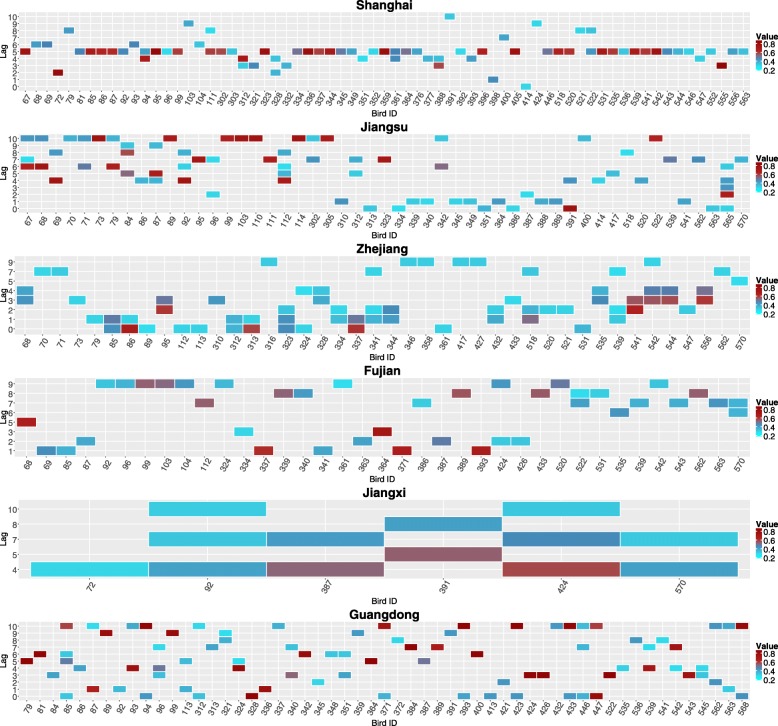
The results of CCF analysis for each bird species with respect to Influenza A (H7N9) cases in Shanghai and other five provinces. The values of correlation coefficients greater than or equal to 0.27 are shown in different colours. Bird species with positive lags *Lag* ≤ 10 are demonstrated in x-axis for each municipality/province, where the lags are measured by weeks

**Fig. 3:**
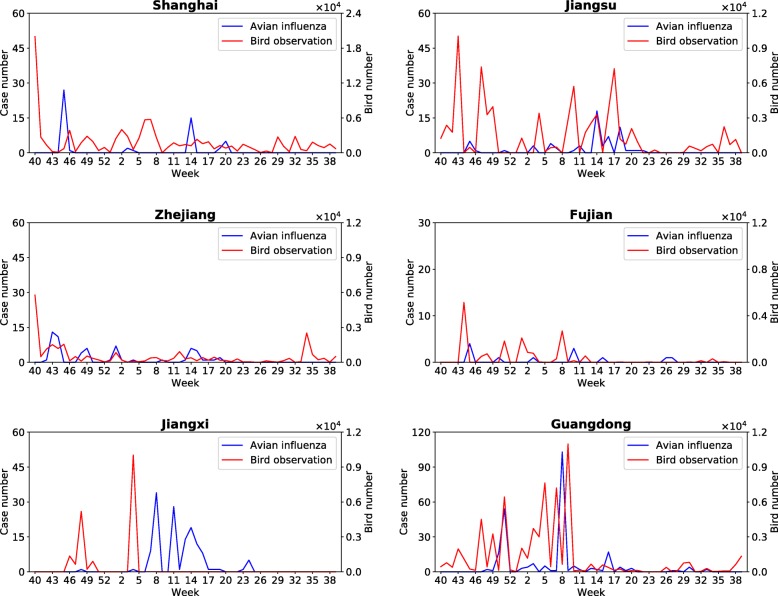
Time series of isolated cases of influenza A (H7N9) and observations of identified migratory birds with positive lags *Lag* ≤ 5 by weeks in Shanghai and other five provinces. Both H7N9 cases and observation data of identified migratory bird species are aggregated by weeks and mapped into one year with respect to their dates of isolation and observation. The x-axis is started from week 40

During the three epidemic waves, the sequences of H7N9 virus have evolved and spread across Southeast China, where migratory birds may play an important role. Accordingly, the CCF analysis is conducted based on 150 common migratory bird species, which are selected with the help of bird experts, to explore their correlations and corresponding lags with respect to the identified H7N9 cases in chicken in China. Figure 2 illustrates the results of CCF analysis for each bird species in Shanghai, Jiangsu, Zhejiang, Fujian, Jiangxi, and Guangdong. Time series of bird observation records with different positive lag values are analysed, where the lags are measured by weeks. The values of correlation coefficients greater than or equal to 0.27 are shown in different colours, while corresponding bird species with positive lags (i.e., *Lag* ≤ 10) are demonstrated in x-axis. In doing so, a list of migratory bird species can be identified for each municipality/province (see Additional file 8), which may be responsible for the introduction of influenza A (H7N9) epidemics in these areas. It can also be observed from Figs. 2 and 3 that the CCF results and temporal distribution of identified bird species in Jiangxi Province are different from other municipality/provinces. The reason is that besides bird migration, poultry trading is also one of the most important reasons for geographical spread of influenza A (H7N9) virus. The result suggests that deeper investigation should be implemented to explain such differences in the future.

**Fig. 4:**
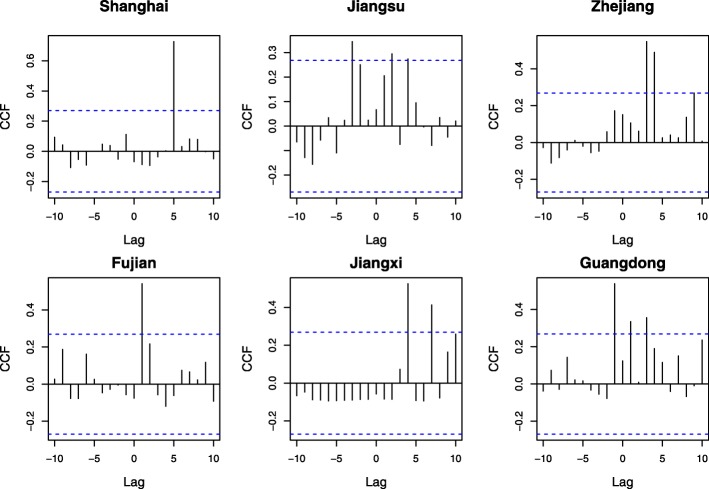
The results of CCF analysis for identified bird species with positive lags *Lag* ≤ 5 in Shanghai and other five provinces. The dotted blue line indicates that the value of correlation coefficient is 0.27. A positive Lag value represents the correlation between the amount of observed bird species at time *t* and that of H7N9 cases at time *t* + *Lag*, where Lag is measured by weeks

The observation records of identified bird species with positive lags (*Lag* ≤ 5) are integrated by weeks with respect to the date of observation in Shanghai and other five provinces. Figure 3 demonstrates both time series of H7N9 cases and observation records of identified bird species, which are mapped into one year starting from week 40. It can be observed that there is a strong correlation between influenza A (H7N9) cases and the amount of identified bird species within five weeks. Further, the CCF analysis is conducted to evaluate the cross correlation between time series of H7N9 cases and identified bird species in each municipality/province. Figure 4 shows the analysis results, where the dotted blue line indicates that the threshold value of correlation coefficient is 0.27. It can be observed that in each municipality/province, there is at least one lag within five weeks with a correlation coefficient greater than the threshold.

**Fig. 5:**
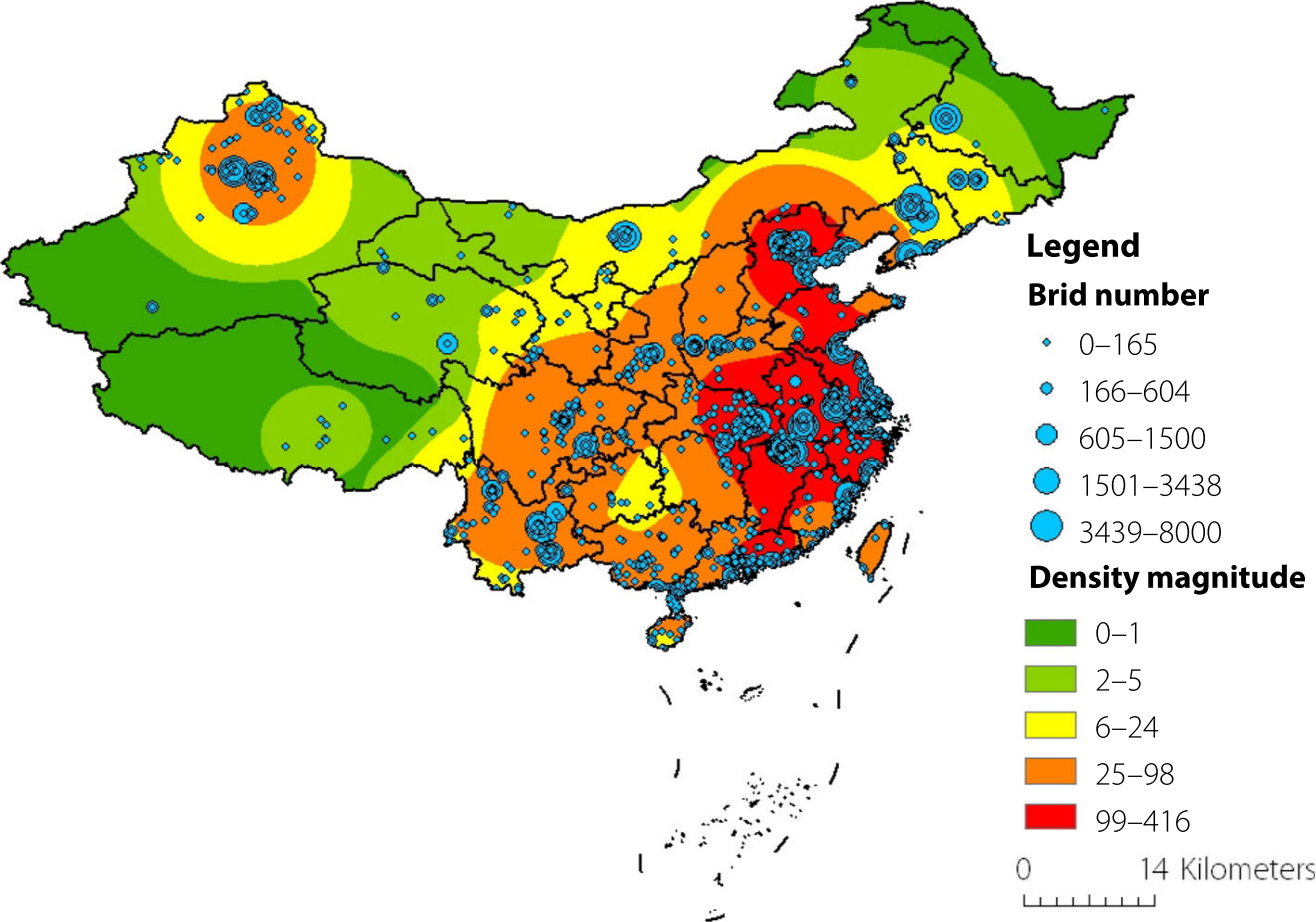
The geographic distribution of identified bird species with positive lags *Lag* ≤ 5 in China. The size of the nodes in blue represents the total number of observed bird species. The coloured surface represents the density magnitude of bird species after smoothing. The map is generated using ArcGIS v.10.5

**Fig. 6:**
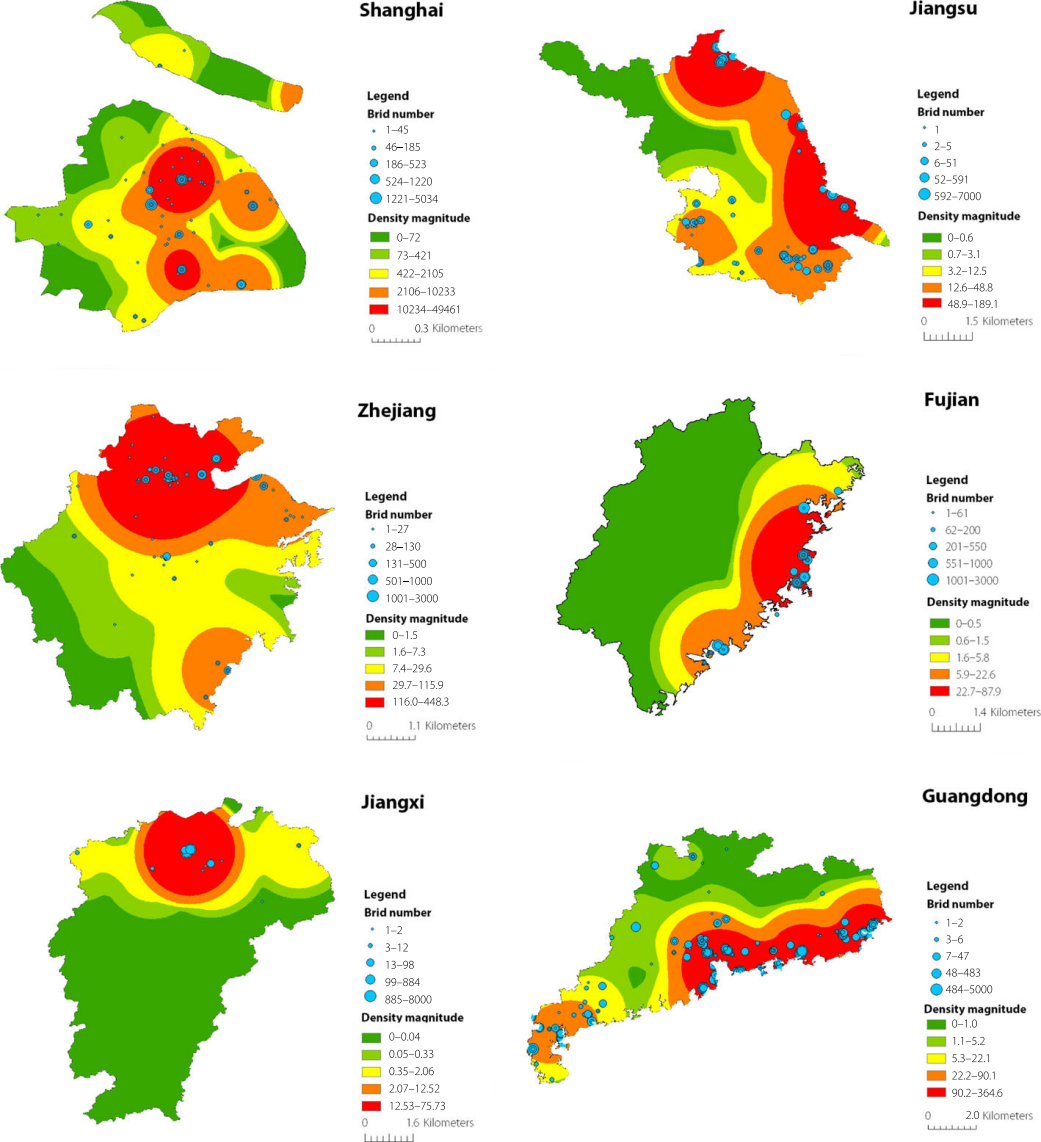
The geographical hotspots of identified bird species with positive lags *Lag* ≤ 5 in Shanghai and other five provinces. The size of nodes in blue represents the total number of identified bird species in each municipality/province. The coloured surface represents the density magnitude of bird species after smoothing. The map is generated using ArcGIS v.10.5

Geographic distribution and kernel density of the identified bird species with positive lags (*Lag* ≤ 5) are illustrated in Fig. 5. The size of nodes in blue represents the number of birds, while the coloured surface represents the density magnitude of bird species after smoothing. In this paper, we focus mainly on the geographical spread of influenza A (H7N9) virus in Eastern China along the East Asian-Australasian flyway. Based on the locality of observation, the geographical hotspots of bird species that are relevant to the introduction of H7N9 virus are further investigated within each municipality/province. Figure 6 illustrates the potential hotspots of H7N9 epidemics based on the geographical distribution of identified migratory bird species in Shanghai, Jiangsu, Zhejiang, Fujian, Jiangxi, and Guangdong. It can be observed that most hotspots are located either along the coastal areas or around large lakes. For example, there are hotspots in Shanghai, Jiangsu, Zhejiang, Fujian, and Guangdong that are along the east coast of China. In Jiangsu Province, there is another hotspot that is around Taihu Lake and close to Wuxi City and Suzhou City. In Jiangxi Province, the major hotspot is located around Poyang Lake.

## Discussion

In order to achieve active surveillance of AIVs in China, it would be critical to systematically identify relevant bird species and their geographical hotspots at a finer-grained scale. In this case, one of the most important issues is to investigate the migratory patterns of various bird species. Traditionally, the bird banding method is widely used by ornithologists to help in keeping track of the movements of bird species and their life history. However, it is very costly and time-consuming to recycle bird rings. Usually, only about 0.3% rings can be recycled. With the development of GPS technologies, the satellite tracking method has been widely used in tracing bird migration pathways [39]. However, because the equipment and signal recovery are very expensive, it cannot be widely applied to monitor a large number of bird species. In recent years, based on the concept of citizen science, it is possible to collect huge amounts of bird observation data from a large-scale area with the help of thousands of bird-watching enthusiasts through crowd-sourcing data collection platforms [40, 41]. For example, the Cornell Lab of Ornithology launched a platform (eBird: https://ebird.org) and several mobile applications to collect and share bird observation data all over the world. Similarly, in China, thousands of bird-watching enthusiasts have spontaneously set up a China Bird Watching Network and shared their observations through the Bird Record Center of China (http://www.birdreport.cn/). Since 2003, the China Ornithological Society has published several annual reports, namely China Bird Report, to share complied and vetted bird records based on birdwatchers’ observations [42]. Accordingly, it is possible for us to analyse the spatiotemporal distribution and migration patterns of bird species at a finer-grained scale and within a larger geographical area [43–46].

Recently, with the development of high-throughput sequencing technology, it is becoming easier to isolate gene sequences of AIVs. The GISAID provides a global platform for sharing avian influenza gene sequences, which are annotated with some additional information, such as the provincial location and the date of isolation. Along this line, many phylogeographic studies have focused on investigating global footprint of influenza A virus (e.g., H5N1) [10, 31–33]. However, because most annotations of AIVs in GISAID do not provide either precise latitude and longitude coordinates of isolation sites or species-specific information (e.g., bird names) associated with avian isolates, phylogeographic analysis can only be implemented at a coarse-grained scale. It has been argued that to promote more through phylogeographic study, it would be better to provide as precise information about avian influenza isolates as possible for GISAID. To make up for such limitations, in this paper, we have identified key migratory birds from 1145 bird species in China. Specifically, the spatiotemporal distribution of identified key bird species can perform as a *prior* for date calibration and location estimation in Bayesian phylogeographic methods [47], which can further help understand the spread of AIVs at a finer-grained scale.

During breeding or winter seasons, many bird species aggregate at favourable stopovers or habitats with high population density, which are critical for transmission of AIVs among different migratory bird species, and between migratory and domestic fowls [17]. Infected migratory birds can then move to other locations, causing new infections elsewhere. In view of this, such gathering locations of bird species are more likely to be the ancestral locations, where reassortment or recombination of different AIVs takes place. In this case, the crowd-sourcing data collection platform for bird species all over China provide a new viewpoint to predict and monitor the spread, recombination, and reemergence of AIVs among migratory birds. Specifically, based on the collected 157 272 observation records of 1145 bird species in China, we have conducted an in-depth investigation on the relationship between the spatiotemporal distribution of migratory birds and historical epidemics of influenza A (H7N9). A list of key bird species as well as their geographical hotspots have been identified for the implementation of active surveillance about influenza A (H7N9) at high epidemic areas. In the future, it is expected to integrate bird observation data in China with more comprehensive records from eBird. With the joint efforts of ornithologists, epidemiologists and molecular biologists, a thorough investigation about AIVs in birds all over the world can be conducted to assess the potential intercontinental movement of influenza A (H7N9) virus, as well as the possible introduction pathway of novel AIVs across continents [48].

It is important to point out that due to the data availability at this moment, there still have several limitations in this paper. First, we only explored the relationship between the spatiotemporal distribution of migratory birds and the spread of influenza A (H7N9) cases in chicken in China. In the future, it would be more meaningful to further explore the relationship between bird migration and the spread of other AIVs (e.g., H5N1). Second, the bird observation data is imbalanced in China. It can be observed from Fig. 5 that there are more observation records along the East Asian–Australasian flyway (i.e., the Eastern China), but relatively fewer observations along the Central Asian flyway (i.e., the Western China). However, it does not indicate that the number of bird species in Eastern China is larger than that in Western China. One possible reason is that peoples in Eastern China have relatively higher income such that they are more likely to be bird-watching enthusiasts. With the help of the citizen science project, it is expected that more bird observation data will be collected in the future. In doing so, the impact of the data imbalance problem should be negligible. Third, in this paper, we only identified geographical hotspots in six municipality/provinces, which account for 91.2% of the total number of isolated H7N9 cases in chicken in GISAID. By unifying both molecular evolution of AIVs and spatial ecology of migratory birds, it would be possible to carry out a systematic analysis on different types of AIVs in China to investigate the risk of newly emerging AIVs through recombination and/or reassortment. To achieve this goal, we make an appeal to colleagues in the study of AIVs that it is better to annotate gene sequences of avian isolates with detailed information (e.g., specific bird names and GPS locations) when uploading to the GISAID database. Finally, it is important to note that although we have identified key bird species and geographical hotspots based on CCF analysis, it does not mean that the H7N9 cases in corresponding locations are introduced by migratory birds. The reason is that poultry trading is also one of the most important reasons for the geographical spread of influenza A (H7N9) virus. In this case, it would be necessary to involve poultry trading data into future analysis.

## Conclusions

In this paper, we have systematically analysed the relationship between geographical spread of influenza A (H7N9) epidemics and spatiotemporal distribution of bird species in China. Specifically, we have identified key bird species and geographical hotspots that are relevant to the introduction of H7N9 epidemics in six major epidemic areas in China (i.e., Shanghai, Jiangsu, Zhejiang, Fujian, Jiangxi, and Guangdong). First, we have conducted phylogenetic analysis on both HA and NA segments of influenza A (H7N9) virus isolated in chicken in China from 2013 to 2017. The reconstructed phylogenetic tree reveals three major epidemic waves in chicken in Eastern China along the East Asian-Australasian flyway of migratory birds. Second, with the help of a citizen science project, we have collected more than 157 272 bird observation records of 1145 bird species all over China using a crowd-sourcing data collection platform. By implementing cross correlation analysis, we have identified the key species from 150 common migratory bird species for each municipality/province, whose temporal distribution are strongly relevant to time series of H7N9 cases within five weeks. Accordingly, we have finally identified potential hotspots of H7N9 epidemics based on the spatial distribution of identified migratory bird species in Shanghai, Jiangsu, Zhejiang, Fujian, Jiangxi, and Guangdong. The findings in this paper would help public health authorities to implement active surveillance and control during the epidemic season of AIVs.

## Additional files

Additional file 1: Multilingual abstracts in the five official working languages of the United Nations. (PDF 175 kb)

Additional file 2: The 495 sequence alignments of influenza A (H7N9) isolated in chicken from GISAID. (FAS 1511 kb)

Additional file 3: IDs and names of selected 150 common migratory birds in China. (XLSX 16 kb)

Additional file 4: 184 gene sequences of influenza A (H7N9) virus isolated in chicken downloaded from GISAID. (XLSX 17 kb)

Additional file 5: The constructed phylogenetic tree based on 184 gene sequences of influenza A (H7N9) virus using MP approach. (EPS 232 kb)

Additional file 6: The constructed phylogenetic tree based on 184 gene sequences of influenza A (H7N9) virus using ML approach. (EPS 234 kb)

Additional file 7: The date-calibrated tree constructed based on 184 gene sequences of influenza A (H7N9) virus. (PDF 38 kb)

Additional file 8: A list of identified bird species in Shanghai and other five provinces. (XLSX 23 kb)

## Abbreviations

AIC: Akaike information criterion
AIV: Avian influenza Virus
CCF: Cross correlation function
GISAID: Global Initiative on Sharing All Influenza Data
HA: Hemagglutinin
ML: Maximum likelihood
MP: Maximum parsimony
NA: Neuraminidase
NJ: Neighbour-joining

## References

[1] Li Q, Zhou L, Zhou M, Chen Z, Li F, Wu H (2014). Epidemiology of human infections with avian influenza a (H7N9) virus in China. New Engl J Med.

[2] Zhou L, Ren R, Yang L, Bao C, Wu J, Wang D (2017). Sudden increase in human infection with avian influenza a (H7N9) virus in China, September-December 2016. Western Pacific Surveillance and Response Journal: WPSAR.

[3] Zhou L, Tan Y, Kang M, Liu F, Ren R, Wang Y (2017). Preliminary epidemiology of human infections with highly pathogenic avian influenza a (H7N9) virus, China, 2017. Emerg Infect Dis.

[4] Su S, Gu M, Liu D, Cui J, Gao GF, Zhou J (2017). Epidemiology, evolution, and pathogenesis of H7N9 influenza viruses in five epidemic waves since 2013 in China. Trends Microbiol.

[5] Alexander DJ (2000). A review of avian influenza in different bird species. Vet Microbiol.

[6] Normile D (2006). Avian influenza: evidence points to migratory birds in H5N1 spread. Science.

[7] Kilpatrick AM, Chmura AA, Gibbons DW, Fleischer RC, Marra PP, Daszak P (2006). Predicting the global spread of H5N1 avian influenza. P Natl Acad Sci USA..

[8] Peterson AT, Benz BW, Papes M (2007). Highly pathogenic H5N1 avian influenza: entry pathways into North America via bird migration. PLoS One.

[9] Liu D, Shi W, Shi Y, Wang D, Xiao H, Li W (2013). Origin and diversity of novel avian influenza a H7N9 viruses causing human infection: phylogenetic, structural, and coalescent analyses. Lancet.

[10] Liang L, Xu B, Chen Y, Liu Y, Cao W, Fang L (2010). Combining spatial-temporal and phylogenetic analysis approaches for improved understanding on global H5N1 transmission. PLoS One.

[11] Shi B, Xia S, Yang GJ, Zhou XN, Liu J (2013). Inferring the potential risks of H7N9 infection by spatiotemporally characterizing bird migration and poultry distribution in eastern China. Infect Dis Poverty.

[12] Wiwanitkit V, Shi B, Xia S, Yang GJ, Zhou XN, Liu J (2013). Research priorities in modeling the transmission risks of H7N9 bird flu. Infect Dis Poverty.

[13] Tian H, Zhou S, Dong L, Van Boeckel TP, Cui Y, Newman SH (2015). Avian influenza H5N1 viral and bird migration networks in Asia. P Natl Acad Sci USA..

[14] Webster RG, Bean WJ, Gorman OT, Chambers TM, Kawaoka Y (1992). Evolution and ecology of influenza a viruses. Microbiol Rev.

[15] Liu Y, Keller I, Heckel G (2011). Range-wide genetic population structure of common pochard (*Aythya ferina*): a potentially important vector of highly pathogenic avian influenza viruses. Ecol Evol.

[16] Webster RG, Yakhno M, Hinshaw VS, Bean WJ, Murti KG (1978). Intestinal influenza: replication and characterization of influenza viruses in ducks. Virology.

[17] Olsen B, Munster VJ, Wallensten A, Waldenström J, Osterhaus ADME, Fouchier RAM (2006). Global patterns of influenza a virus in wild birds. Science.

[18] Pawar S, Pande S, Jamgaonkar A, Koratkar S, Pal B, Raut S (2009). Avian influenza surveillance in wild migratory, resident, domestic birds and in poultry in Maharashtra and Manipur, India, during avian migratory season 2006-07. Curr Sci India.

[19] Bi Y, Zhang Z, Liu W, Yin Y, Hong J, Li X, et al. Highly pathogenic avian influenza A(H5N1) virus struck migratory birds in China in 2015. Sci Rep. 2015;5:12986. 10.1038/srep12986 PMC4531313 26259704

[20] Felsenstein J (1981). Evolutionary trees from DNA sequences: a maximum likelihood approach. J Mol Evol.

[21] Yang Z (1994). Maximum likelihood phylogenetic estimation from DNA sequences with variable rates over sites: approximate methods. J Mol Evol.

[22] Drummond AJ, Rambaut A. BEAST: Bayesian evolutionary analysis by sampling trees. BMC Evol Biol. 2007;7:214. 10.1186/1471-2148-7-214 PMC2247476 17996036

[23] Hall BG (2013). Building phylogenetic trees from molecular data with MEGA. Mol Biol Evol.

[24] Kingman JFC (1982). On the genealogy of large population. J Appl Probab.

[25] Wakeley J (2009). Coalesent theory: an introduction.

[26] Pybus OG, Rambaut A, Harvey PH (2000). An integrated framework for the inference of viral population history from reconstructed genealogies. Genetics.

[27] Drummond AJ, Nicholls GK, Rodrigo AG, Solomon W (2002). Estimating mutation parameters, population history and genealogy simultaneously from temporally spaced sequence data. Genetics.

[28] Opgen-Rhein R, Fahrmeir L, Strimmer K. Inference of demographic history from genealogical trees using reversible jump Markov chain Monte Carlo. BMC Evol Biol. 2005;5(1):6. 10.1186/1471-2148-5-6 PMC548300 15663782

[29] Drummond AJ, Rambaut A, Shapiro B, Pybus OG (2005). Bayesian coalescent inference of past population dynamics from molecular sequences. Mol Biol Evol.

[30] Minin VN, Bloomquist EW, Suchard MA (2008). Smooth skyride through a rough skyline: Bayesian coalescent-based inference of population dynamics. Mol Biol Evol.

[31] Wallace RG, HoDac HM, Lathrop RH, Fitch WM (2007). A statistical phylogeography of influenza a H5N1. P Natl Acad Sci USA..

[32] Gilbert M, Xiao X, Pfeiffer DU, Epprecht M, Boles S, Czarnecki C, Chaitaweesub P, Kalpravidh W, Minh PQ, Otte MJ, Martin V, Slingenbergh J (2008). Mapping H5N1 highly pathogenic avian influenza risk in Southeast Asia. P Natl Acad Sci USA.

[33] Li R, Jiang Z, Xu B (2014). Global spatiotemporal and genetic footprint of the H5N1 avian influenza virus. Int J Health Geogr.

[34] Thompson JD, Gibson TJ, Plewniak F, Jeanmougin F, Higgins DG (1997). The CLUSTAL X windows interface: flexible strategies for multiple sequence alignment aided by quality analysis tools. Nucleic Acids Res.

[35] Hall TA, et al. BioEdit: a user-friendly biological sequence alignment editor and analysis program for Windows 95/98/NT. In Nucleic Acids Symposium Series, Volume 41, [London]: Information Retrieval Ltd., c1979-c2000. 1999:95–98.

[36] Tamura K, Stecher G, Peterson D, Filipski A, Kumar S (2013). MEGA6: molecular evolutionary genetics analysis version 6.0. Mol Biol Evol.

[37] Posada D (2008). jModelTest: phylogenetic model averaging. Mol Biol Evol.

[38] Liu Y, Paquette SG, Zhang L, Leon AJ, Liu W, Xiuming W (2015). The third wave: H7N9 endemic reassortant viruses and patient clusters. J Infect Dev Countr.

[39] Tang M, Zhou Y, Li J, Wang W, Cui P, Hou Y (2011). Exploring the wild birds’ migration data for the disease spread study of H5N1: a clustering and association approach. Knowl Inf Syst.

[40] Sullivan BL, Wood CL, Iliff MJ, Bonney RE, Fink D, Kelling S (2009). eBird: a citizen-based bird observation network in the biological sciences. Biol Conserv.

[41] Hampton SE, Strasser CA, Tewksbury JJ, Gram WK, Budden AE, Batcheller AL (2013). Big data and the future of ecology. Front Ecol Environ.

[42] Li X, Liang L, Gong P, Liu Y, Liang F (2013). Bird watching in China reveals bird distribution changes. Chin Sci Bull.

[43] Tang M, Zhou Y, Cui P, Wang W, Li J, Zhang H, et al. Discovery of migration habitats and routes of wild bird species by clustering and association analysis. In International Conference on Advanced Data Mining and Applications. 2009:288–301.

[44] Fink D, Damoulas T, Dave J. Adaptive Spatio-temporal exploratory models: hemisphere-wide species distributions from massively crowdsourced eBird data. In Twenty-Seventh AAAI Conference on Artificial Intelligence. 2013:1284–90.

[45] Fink D, Damoulas T, Bruns NE, La Sorte FA, Hochachka WM, Gomes CP (2014). Crowdsourcing meets ecology: hemisphere-wide spatiotemporal species distribution models. AI Mag.

[46] Zhan X, Ye Y, Zhuo Y, Shi B, Ren Y, Hu W (2017). Spatial-temporal analysis on bird habitat discovery in China. International Conference on Security, Pattern Analysis, and Cybernetics (SPAC), IEEE.

[47] Lemey P, Rambaut A, Drummond AJ, Suchard MA (2009). Bayesian phylogeography finds its roots. PLoS Comput Biol.

[48] Miller R, Sweeney S, Akkina J, Saito E (2015). Potential intercontinental movement of influenza a (H7N9) virus into North America by wild birds: application of a rapid assessment framework. Transbound Emerg Dis.

