# Supplementary material for: Identifying key bird species and geographical hotspots of avian influenza A (H7N9) virus in China
Source: Infect Dis Poverty. 2018 Oct 11;7:97. doi: 10.1186/s40249-018-0480-x (PMC6180610; doi:10.1186/s40249-018-0480-x)
Supplement: Supplementary file 1 — Multilingual abstracts in the five official working languages of the United Nations. (PDF 175 kb) [file 40249_2018_480_MOESM1_ESM.pdf]

Translation of the abstract into the five official working languages of the United Nations

تحديد الأنواع الرئيسية من الطيور والبؤر الجغرافية لفيروس إنفلونزا الطيور A (H7N9) في الصين

بن يون شي ، وشياو مينغ زان ، وجين شين تشنغ ، وهونغ جون كيو ، وليانغ، ودان ليانغ، ويان مينغ يي ، وقوه جينغ يانغ ، ويانغ ليو ، وجيمنغ ليو

خلفية الموضوع: منذ الإصابة البشرية الأولى التي تم تحديدها في الصين خلال شهري فبراير ومارس 2013 ، تسبب فيروس إنفلونزا الطيور A) (H7N9 في شواغل خطيره في مجال الصحة العامة بسبب انتشاره الواسع وارتفاع معدل الوفيات. وتبين الأدلة أن هجرة الطيور تلعب دورا أساسيا في الانتشار العالمي لفيروسات إنفلونزا الطيور. وبناء على ذلك ، فإننا نهدف في هذا البحث إلى تحديد أنواع الطيور الرئيسية والبؤر الجغرافية ذات الصلة بانتقال فيروس إنفلونزا الطيور A (H7N9) في الصين.
الأساليب: أولا نجري تحليلا وراثيا على 626 من المتواليات الفيروسية لفيروس إنفلونزا الطيور A((H7N9 المعزول في الدجاج ، والتي يتم جمعها من المبادرة العالمية لتبادل بيانات أنفلونزا الطيور (GISAID)، للكشف عن الانتشار الجغرافي والتطور الجزيئي الفيروس في الصين. ثم نعتمد وظيفة الارتباط المتبادل لاستكشاف العلاقة بين الحالات المحددة للإنفلونزا A (H7N9) والتوزيع المكاني والزمني للطيور المهاجرة. وهنا يتم تحديد التوزيع المكاني والزماني لأنواع الطيور استنادا إلى بيانات مراقبة الطيور التي تم جمعها من تقارير الطيور الصينية، والتي تتكون من 157 272 سجل للمراقبة حول 1145 نوع من الطيور. وأخيرا، نقوم باستخدام مقدر كثافة النواة لتحديد النقاط الجغرافية الساخنة لمواطن/محطات توقف الطيور التي لها صله بعدوى الإنفلونزا A (H7N9).
النتائج: يكشف التحليل الوراثي الأنماط التطورية والجغرافية للإصابة بالإنفلونزا A (H7N9)، حيث تتجمع الحالات في نفس البلدية أو المقاطعات القريبة مع اختلافات تطورية صغيره. وعلاوة على ذلك ، فإن ثلاث موجات وبائية في الدجاج على طول طريق التحليق في شرق اسيا-الاسترالية في الصين تختلف عن الشجرة الوراثية. ويحدد تحليل الأطر أنواع الطيور المهاجرة المحتملة ذات الصلة بعدوى الإنفلونزا A (H7N9) في شانغهاي ، وجيانغسو ، وتشجيانغ ، وفوجيان ، وجيانغشي ، وقوانغدونغ في الصين ، حيث تتعامل ستة بلديات/مقاطعات مع 91.2 بالمئة من العدد الإجمالي لحالات ال(H7N9) المنعزلة في الدجاج في مبادرة تبادل بيانات انفلونزا الطيور (GISAID). واستنادا إلى التوزيع المكاني لأنواع الطيور المحددة، فإنه من المقدر والموضح أن تكون البؤر الجغرافية عادة ضمن هذه البلدية/المقاطعات.
الاستنتاجات: يمكن للأنواع التي تم تحديدها من الطيور والبؤر الجغرافية أن توفر الإشارات والأدلة الإنذارية للمراقبة النشطة، بالإضافة إلى التحكم الاستراتيجي بانتقال انفلونزا A(H7N9) في الصين.

Translated from English version into Arabic by Shefa'a Qudah and Madawi Alajmi, through

TRANSLATORS
WITHOUT BORDERS

中国甲型禽流感(H7N9)病毒相关鸟种及热点地区之鉴别

Benyun Shi, Xiao-Ming Zhan, Jin-Xin Zheng, Hongjun Qiu, Dan Liang, Yan-Ming Ye, Guo-Jing Yang, Yang Liu and Jiming Liu

引言：自 2013 年 2 月至 3 月中国首次发现人类感染以来，甲型禽流感(H7N9)病毒以其广泛的传播和高死亡率而引起了公众对健康密切关注。有证据表明，鸟类迁徙在禽流感病毒的全球传播中起着至关重要的作用。因此，本文旨在找出与 H7N9 禽流感病毒在中国传播有关的主要鸟类种及其热点地区。

方法：我们首先对从 GISAID 收集到的 626 个 H7N9 禽流感病毒的基因序列进行了系统发育学分析，揭示了其在中国的空间传播和分子进化模式。然后，我们采用交叉相关函数(CCF)分析了 H7N9 病例与候鸟时空分布的关系。在此，鸟类的时空分布是基于《中国鸟类报告》中针对 1,145 个鸟种的 157,272 条记录生成的。最后，我们使用核密度方法识别出与 H7N9 禽流感疫情相关的鸟类栖息地或中途停留地的热点区域。
结果：系统发育学分析揭示了 H7N9 禽流感的分子进化和空间传播模式，即同一或邻近省市的病例在进化树上有聚集效应，其进化差异很小。在此基础上，我们可以观察到在 East Asian–Australasian 候鸟迁徙路径上，爆发过三波禽流感。进一步针对上海、江苏、浙江、福建、江西和广东六个省市的 CCF 分析，我们识别出可能与 H7N9 感染有关的鸟种。最后，我们根据相关鸟类的空间分布情况对与 H7N9 疫情相关的热点地区做了进一步评估。
结论：本文识别出与 H7N9 传播相关的关键鸟种和热点地区可为我国甲型禽流感疫情的主动监测和控制提供科学依据。

Translated from English version into Chinese by Ben-Yun Shi

Identification des espèces principales d'oiseaux et des zones géographiques sensibles au virus de la grippe aviaire A (H7N9) en Chine

Ben-Yun Shi, Xiao-Ming Zhan, Jin-Xin Zheng, Hong-Jun Qiu, Dan Liang, Yan-Ming Ye, Guo-Jing Yang, Yang Liu et Jiming Liu

Contexte : Depuis la première infection humaine enregistrée en Chine entre février et mars 2013, le virus de la grippe aviaire A (H7N9) a causé de graves problèmes de santé publique en raison de sa propagation et de son taux de mortalité élevé. Les preuves montrent que la migration des oiseaux joue un rôle essentiel dans la propagation mondiale du virus de la grippe aviaire. Par conséquent, dans cet article, nous cherchons à identifier les espèces principales d'oiseaux et les zones géographiques sensibles et pertinentes dans la transmission du virus de la grippe aviaire A (H7N9) en Chine.
Méthodes: Dans un premier temps, une analyse phylogénétique est effectuée sur 626 séquences virales du virus de la grippe aviaire A (H7N9) isolées chez le poulet, collectées dans le cadre de Global Initiative on Sharing Avian Influenza Data (Initiative mondiale pour le partage des données sur la grippe, GISAID), afin de dévoiler l'étendue géographique et l'évolution moléculaire du virus en Chine. Ensuite, nous adoptons la fonction d'intercorrélation (CCF) afin d'analyser la relation entre les cas de grippe identifiés A (H7N9) et la répartition spatio-temporelle des oiseaux migrateurs. Dans ce cas, la répartition spatio-temporelle des espèces d'oiseaux est générée sur la base des données d'observation d'oiseaux, recueillies par China Bird Reports, qui comprend 157 272 observations sur 1145 d'espèces d'oiseaux. Enfin, un estimateur de densité de noyau est utilisé afin d'identifier les zones géographiques sensibles de l'habitat / de l'escale des oiseaux qui sont pertinentes pour les infections de la grippe A (H7N9).
Résultats: L'analyse phylogénétique révèle les profils évolutifs et géographiques des infections de la grippe A (H7N9), où des cas, dans une même municipalité/province ou voisines, sont regroupés, révélant des différences évolutives minimes. De plus, trois vagues épidémiques affectant les poulets le long de la voie de migration Asie de l'Est-Australasie en Chine se distinguent de l'arbre

phylogénétique. L'analyse CCF identifie les espèces d'oiseaux migrateurs pertinentes aux infections de la grippe A (H7N9) à Shanghai, Jiangsu, Zhejiang, Fujian, Jiangxi et Guangdong en Chine, où les six municipalités / provinces représentent 91,2 % du nombre total de cas isolés H7N9 chez le poulet dans le cadre de GISAID. Basé sur la répartition spatiale des espèces d'oiseaux identifiées, les zones géographiques sensibles sont, en outre, estimées et illustrées au sein de ces communes / provinces typiques.
Conclusions: Les espèces d'oiseaux et les zones géographiques sensibles identifiées, pourraient fournir un signal sentinelle et des preuves pour une surveillance active, ainsi qu'un contrôle stratégique de la transmission de la grippe A (H7N9) en Chine.

Translated from English version into French by Alice Montergnole and Eve Anderson, through

TRANSLATORS
WITHOUT BORDERS

Выявление главных видов птиц и географических очагов птичьего гриппа A(H7N9) в Китае.

Бэнь-Юнь Ши, Сяо-Мин Чжань, Цзинь-Синь Чжэн, Хун-Цзюнь Цю, Дань Лян, Янь-Мин Е, Го-Цзин Ян, Ян Лю и Цзимин Лю

Краткое описание: С тех пор, как в Китае в феврале и марте 2013 года были зарегистрированы первые случаи заражения людей, вирус птичьего гриппа A(H7N9) представляет собой серьезную проблему для общественного здравоохранения вследствие его широкого распространения и высокого уровня смертности. Факты показывают, что миграция птиц играет существенную роль в глобальном распространении вирусов птичьего гриппа. В этой связи, в данной статье мы стремимся определить главные виды птиц и географические очаги птичьего гриппа A(H7N9) в Китае.
Методы: Сначала мы выполнили филогенетический анализ 626 последовательностей геномов вируса птичьего гриппа A(H7N9), выделенных у куриц, которые были выбраны из базы данных Глобальной инициативы по обмену данными о гриппе (GISAID), чтобы установить географическое распределение и молекулярную эволюцию вируса в Китае. Затем с помощью функции взаимной корреляции (CCF) мы исследовали отношение между обнаруженными случаями заболевания гриппом A(H7N9) и пространственно-временным распределением мигрирующих птиц. Пространственно-временное распределение видов птиц создавалось на основании данных, собранных из отчетов наблюдения за птицами в Китае, которые состоят из 157 272 записей об 1145 видах птиц. В конце мы сделали оценку ядерной плотности для определения географических очагов обитания и стоянок птиц, имеющих отношение к случаям заражения гриппом A(H7N9).
Результаты: Филогенетический анализ выявил эволюционные и географические закономерности заражения гриппом A(H7N9), при которых случаи заболевания с небольшими эволюционными различиями были сконцентрированы в одном и том же или

близлежащем муниципальном округе/провинциях. Более того, три волны эпидемий у куриц вдоль восточного азиатско-австралийского миграционного пути в Китае отличались от филогенетического дерева. С помощью анализа CCF были определены возможные виды мигрирующих птиц, которые имеют отношение к инфицированию гриппом A(H7N9) в Шанхае, Цзянсу, Чжэцзяне, Фуцзяне, Цзянси и Гуандуне в Китае, где по данным GISAID в шести муниципальных округах/провинциях насчитывается 91,2 % от общего числа случаев выявления у куриц вируса H7N9. На основании пространственного распределения идентифицированных видов птиц в пределах этих типичных муниципальных округов/провинций была произведена дальнейшая оценка и наглядная демонстрация географических очагов.

Выводы: Идентифицированные виды птиц и географические очаги могут служить сигнальными индикаторами и обеспечивать данные для активного наблюдения, а также для стратегического контроля над передачей вируса гриппа A(H7N9) в Китае.

Translated from English version into Russian by Natalia Potashnik and Liudmila Tomanek, through

TRANSLATORS
WITHOUT BORDERS

Identificación de las especies principales de aves y de los puntos geográficos críticos con respecto al virus de la gripe aviar A (H7N9) en China

Ben-Yun Shi, Xiao-Ming Zhan, Jin-Xin Zheng, Hong-Jun Qiu, Dan Liang, Yan-Ming Ye, Guo-Jing Yang, Yang Liu y Jiming Liu

Antecedentes: Desde que se identificaron las primeras infecciones en humanos en China durante los meses de febrero y marzo de 2013, el virus de la gripe aviar A (H7N9) ha sido motivo de grave preocupación en materia de salud pública debido a su gran propagación y a su alta tasa de mortalidad. Las pruebas demuestran que la migración de las aves tiene un papel fundamental en la propagación de los virus de la gripe aviar. Por lo tanto, este artículo tiene como objetivo identificar las especies principales de aves y los puntos geográficos críticos que son pertinentes con respecto a la transmisión del virus de la gripe aviar A (H7N9) en China.

Métodos: En primer lugar, se lleva a cabo un análisis filogenético de 626 secuencias virales del virus de la gripe aviar A (H7N9) aisladas en pollos obtenidas de la Iniciativa Global para Compartir Datos sobre la Gripe Aviar (GISAID, su sigla en inglés) a fin de revelar la propagación geográfica y la evolución molecular del virus en China. Luego, aplicamos la función de correlación cruzada (FCC) para analizar la relación entre los casos identificados de gripe aviar A (H7N9) y la distribución espacio-temporal de las aves migratorias. En este aspecto, la distribución espacio-temporal de las aves migratorias se obtiene de los datos de observación de las aves recopilados en los Informes sobre Aves de China, que se compone de 157.272 registros de observación sobre 1.145 especies de aves. Por último, se aplica el método Kernel para estimar la densidad a fin de identificar los puntos geográficos críticos de hábitat o parada de las aves que son pertinentes con respecto a las

infecciones por gripe A (H7N9).

Resultados: El análisis filogenético revela los patrones evolutivos y geográficos de las infecciones por gripe A (H7N9), donde los casos en el mismo municipio o provincia, o en municipios o provincias cercanos, se agrupan con diferencias evolutivas mínimas. Es más, en el árbol filogenético se distinguen tres olas epidémicas en los pollos a lo largo de la ruta migratoria de Asia Oriental-Australasia en China. El análisis FCC identifica las especies posibles de aves migratorias que son pertinentes con relación a las infecciones por gripe A (H7N9) en Shanghai, Jiangsu, Zheijiang, Fujian, Jiangxi y Guangdong, en China, donde estos seis municipios o provincias dan cuenta del 91,2% del número total de casos de H7N9 aislados en pollos registrados en la GISAID. Sobre la base de la distribución espacial de las especies de aves identificadas, los puntos geográficos críticos a su vez se estiman y se ilustran dentro de estos municipios o provincias típicos.

Conclusiones: Las especies de aves identificadas y los puntos geográficos críticos podrían proporcionar señales y pruebas centinelas para llevar a cabo una vigilancia activa, así como un control estratégico de la transmisión de la gripe A (H7N9) en China.

Translated from English version into Spanish by Ribcamar and Syl Figu, through

TRANSLATORS
WITHOUT BORDERS
