# Supplementary figures and images for: Identifying key bird species and geographical hotspots of avian influenza A (H7N9) virus in China
Source: Infect Dis Poverty. 2018 Oct 11;7:97. doi: 10.1186/s40249-018-0480-x (PMC6180610; doi:10.1186/s40249-018-0480-x)

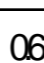

Supplement: Supplementary file 7 — The date-calibrated tree constructed based on 184 gene sequences of influenza A (H7N9) virus. (PDF 38 kb) [file 40249_2018_480_MOESM7_ESM.pdf]
